# The mechanisms of hyphal pellet formation mediated by polysaccharides, α-1,3-glucan and galactosaminogalactan, in *Aspergillus* species

**DOI:** 10.1186/s40694-020-00101-4

**Published:** 2020-07-01

**Authors:** Ken Miyazawa, Akira Yoshimi, Keietsu Abe

**Affiliations:** 1 Laboratory of Applied Microbiology, Department of Microbial Biotechnology, Graduate School of Agricultural Science, Tohoku University, 468-1 Aramaki-Aoba, Aoba-ku, Sendai, 980-8572 Japan; 2 Laboratory of Environmental Interface Technology of Filamentous Fungi, Graduate School of Agriculture, Kyoto University, Oiwake-cho, Kitashirakawa, Sakyo-ku, Kyoto, 606-8502 Japan; 3 ABE-project, New Industry Creation Hatchery Center, Tohoku University, 6-6-10 Aramaki-Aoba, Aoba-ku, Sendai, 980-8579 Japan; 4 Laboratory of Microbial Resources, Department of Microbial Biotechnology, Graduate School of Agricultural Science, Tohoku University, 468-1 Aramaki-Aoba, Aoba-ku, Sendai, 980-8572 Japan

**Keywords:** Hyphal aggregation, Filamentous fungi, Cell wall, α-1,3-Glucan, Galactosaminogalactan

## Abstract

Filamentous fungi are widely used for production of enzymes and chemicals, and are industrially cultivated both in liquid and solid cultures. Submerged culture is often used as liquid culture for filamentous fungi. In submerged culture, filamentous fungi show diverse macromorphology such as hyphal pellets and dispersed hyphae depending on culture conditions and genetic backgrounds of fungal strains. Although the macromorphology greatly affects the productivity of submerged cultures, the specific cellular components needed for hyphal aggregation after conidial germination have not been characterized. Recently we reported that the primary cell wall polysaccharide α-1,3-glucan and the extracellular polysaccharide galactosaminogalactan (GAG) contribute to hyphal aggregation in *Aspergillus oryzae*, and that a strain deficient in both α-1,3-glucan and GAG shows dispersed hyphae in liquid culture. In this review, we summarize our current understanding of the contribution of chemical properties of α-1,3-glucan and GAG to hyphal aggregation. Various ascomycetes and basidiomycetes have α-1,3-glucan synthase gene(s). In addition, some Pezizomycotina fungi, including species used in the fermentation industry, also have GAG biosynthetic genes. We also review here the known mechanisms of biosynthesis of α-1,3-glucan and GAG. Regulation of the biosynthesis of the two polysaccharides could be a potential way of controlling formation of hyphal pellets.

## Introduction

Over 1.5 million species of filamentous fungi inhabit the earth and play a central role in global material circulation as decomposers [1]. As filamentous fungi are well adapted to markedly diverse habitats, many species are used in industry, e.g. to produce enzymes and chemicals, due to their varied metabolic capabilities [2–4]. The industrial production is commonly performed by liquid cultivation of fungi, which makes it easy to supply nutrients and induce gene expression. The scale of industrial fungal cultures sometimes reaches dozens of tonnes. In submerged cultivation, filamentous fungi grow in various macromorphological forms, such as pellets and pulp, depending on agitation speed, pH, medium composition, and other conditions, and also on the genetic backgrounds of fungal strains [4, 5]. Macromorphology greatly affects the productivity of submerged cultivation [2].

The diameters of hyphal pellets can reach several millimeters, which limits oxygen and nutrient transfer to the inner region of the pellets [6] because oxygen and nutrients can reach only 200 µm from the pellet surface in submerged culture [7]. Culture broth containing fungal pellets shows Newtonian flow behavior from the rheological viewpoint, whereas dispersed hyphae sometimes cause high viscosity and consequently decrease the efficiency of mixing [5]. The non-Newtonian property of culture medium containing dispersed hyphae limits convective oxygen transport [4]. The preferred macromorphology of filamentous fungi in liquid culture depends on the target product. For instance, pellet form is preferable for production of citric acid by *Aspergillus niger* [8, 9], whereas dispersed hyphae are suitable for polygalacturonidase and glucoamylase production by *A. niger* [6, 10, 11] and α-amylase production by *Aspergillus oryzae* [12].

Understanding the mechanism of pellet formation in submerged cultures of filamentous fungi is important for industrial production. Prior to hyphal aggregation, conidial aggregation often takes place [13, 14]. Conidia of various filamentous fungi are coated with hydrophobins and melanins, which cause conidial aggregation via hydrophobic and electrostatic interactions, respectively [14]. The mechanism of conidial aggregation is well studied and has been covered in detail elsewhere [14, 15]. In this review, we focus on hyphal aggregation after germination. When the dormancy of conidia is broken and the conidia are swollen, the melanin and hydrophobin layer is broken, and the cell wall polysaccharides become exposed on the cell surface [14]. Here we overview the cell wall components contributing to the formation of hyphal pellets in filamentous fungi, especially in *Aspergillus* species.

The fungal cell wall, a complex and highly dynamic structure, is composed mainly of polysaccharides. In *Aspergillus* species, these polysaccharides are mostly α-glucans (α-1,3-glucan with a small amount of α-1,4-glucan), β-glucans (β-1,3-glucan with 1,6-branches), chitin, and galactomannan [16–18]. The cell wall also contains galactomannoproteins and glycosylphosphatidylinositol (GPI)-anchored proteins [19, 20]. Some fungi have an extracellular matrix (ECM), which is composed of polysaccharides, proteins, lipids, and nucleic acids [19, 21, 22], and is associated with (or attached to) the major cell wall polysaccharides [17, 19, 23]. The ECM composition varies among fungal species [17]. In *Aspergillus* species, the surface of hyphal cell is decorated with the ECM material mainly composed of galactosaminogalactan (GAG), α-1,3-glucan, and galactomannan [24, 25]. In 2010, Fontaine et al. [26] reported that α-1,3-glucan plays a role in conidial aggregation during swelling in *Aspergillus fumigatus*. Through functional analysis of α-1,3-glucan synthase genes in *Aspergillus nidulans*, we found that the hyphae of an α-1,3-glucan-deficient strain are dispersed under liquid culture conditions with shaking, whereas wild-type mycelia form pellets [27]. We further revealed that, in addition to α-1,3-glucan, the extracellular secreted polysaccharide GAG contributes to the formation of hyphal pellets in the industrially used fungus *A. oryzae* [28]. This review describes the proposed mechanism of fungal pellet formation mediated by biochemical properties of cell wall polysaccharides, in particular α-1,3-glucan and GAG in *Aspergillus* species, and outlines the potential for controlling pellet formation by regulating the biosynthesis of the polysaccharides.

## Hyphal aggregation factors in filamentous fungi: biological functions and biochemical properties

Hyphal aggregation in filamentous fungi is thought to be affected by the properties of hyphal surface components. The surface of a hyphal cell is covered with the cell wall, which has essential roles in survival (i.e., maintaining the cell shape and shielding the cells from environmental stresses). The cell wall of fungi is the first component to make contact with host cells, substrates, and themselves (hyphal aggregation) [16]. Understanding the cell wall structure is essential for the control of fungal pathogenesis, industrial productivity, and so on. We recently reported that α-1,3-glucan and GAG contribute to hyphal aggregation [28], and a study of the mechanism of hyphal aggregation mediated by the polysaccharides is ongoing. In this section, we review the biological functions of the polysaccharides related to hyphal aggregation and the mechanism of hyphal aggregation in liquid culture.

### α-1,3-Glucan

By the early 1970s, α-1,3-glucan was known to be present in the cell wall of fungi such as *A*. *niger*, *Cryptococcus neoformans*, *Schizosaccharomyces pombe*, *Polyporus* species, and *Paracoccidioides brasiliensis* [29–32], but the biological function of α-1,3-glucan was poorly understood. In the 1970s, Zonneveld [33–37] found that α-1,3-glucan has a role of a reserve polysaccharide, and analyzed in detail its function related to the cell wall and cleistothecia development in *A*. *nidulans*. San-Blas et al. [38–41] first mentioned the relationship between α-1,3-glucan and pathogenesis in *P*. *brasiliensis* in 1976. After Klimpel and Goldman [42, 43] had suggested α-1,3-glucan as a pathogenic factor in *Histoplasma capsulatum*, similar biological function of α-1,3-glucan was reported in *Blastomyces dermatitidis* and *C*. *neoformans* [44, 45]. Direct evidence that α-1,3-glucan contributes to pathogenesis in *H*. *capsulatum* was reported in 2004 [46]. In *H*. *capsulatum*, α-1,3-glucan is located on the surface of the cell wall and interferes with the recognition of the fungal cells by β-glucan receptors on host phagocytic cells [47]. After the whole genome analysis of many fungi, α-1,3-glucan synthase genes were annotated in numerous fungal species, and the functional studies of these genes were pioneered in *A. fumigatus* [48–51]. A disruption strain of *A*. *fumigatus* lacking all three α-1,3-glucan synthase genes (Δ*ags*) lost α-1,3-glucan from the cell wall [50] and showed decreased pathogenesis in a murine aspergillosis model [51]. Cryptococcal α-1,3-glucan is required for the association of the capsule with cells, and the absence of α-1,3-glucan resulted in capsule disassembly and avirulence in a mouse model [52, 53]. In the rice blast fungus *Pyricularia oryzae* (*Magnaporthe grisea*), the surface of infectious hyphae is covered with α-1,3-glucan, which contributes to resistance to cell wall–digesting enzymes such as β-1,3-glucanase and chitinase, resulting in inhibition of the release of pathogen-associated molecular patterns from hyphae and in infection of the host cells [54, 55]. On the other hand, α-1,3-glucan itself induces maturation of human dendritic cells [56], meaning that further research is needed to elucidate the contribution of α-1,3-glucan to virulence. Another function of α-1,3-glucan is inhibition of adsorption of α-amylase to cell wall chitin in *A*. *oryzae* [57, 58]; under poor nutrient conditions, cell wall α-1,3-glucan is degraded to supply glucose to hyphal cells, and then secreted α-amylase adsorbs to chitin exposed in the cell wall, which might contribute to efficient hydrolysis of starch and subsequent sugar uptake by the cells. Bacterial α-1,3-glucan in sticky dental plaque is an inducer substrate of mutanase, which degrades the plaque; mutanase is primarily produced by the *Trichoderma* genus [59]. α-1,3-Glucan derived from *Laetiporus sulphureus* [60] and *Cerrena unicolor* [59] is also a mutanase synthesis inducer. Antitumor activity of fungal α-1,3-glucan [61, 62] and more recently its potential use as a biosorbent for heavy metals [63] have been reported. The heat resistance of ester derivatives of α-1,3-glucan is higher than that of thermoplastics such as polyethylene terephthalate or nylon 6 [64, 65]. Taken together, the evidence available today indicates that α-1,3-glucan has various biological functions, such as being a virulence factor in some pathogenic fungi, and studies on the potential application of α-1,3-glucan as an industrial material are ongoing.

In 2010, Fontaine et al. [26] reported that α-1,3-glucan contributes to aggregation of germinating conidia of *A*. *fumigatus*. Treatment of germinating conidia with α-1,3-glucanase prevents the aggregation. In addition, α-1,3-glucan-coated latex beads adhere to the surface of swollen conidia and mutan. These results demonstrate that α-1,3-glucan plays an essential role for aggregation [26]. The hyphae of *A*. *nidulans* form pellets in shake-flask culture [27, 66]. Yoshimi et al. [27] first reported that the disruption of the α-1,3-glucan synthase gene *agsB* in *A*. *nidulans* resulted in a cell wall lacking α-1,3-glucan and in dispersed hyphae in submerged culture. He et al. [66] reported that hyphae formed smaller pellets in the *agsB* disruptant in shaken liquid culture in comparison with the parental strain. Formation of small pellets by the *agsB* disruptant of He et al. [66] but not by that of Yoshimi et al. [27] might be explained by the difference in the genetic backgrounds of the two disruptants; for instance, the former might produce a larger amount of another hyphal aggregation factor, GAG (see Subsection “Galactosaminogalactan”, than the latter. These results suggest that α-1,3-glucan is a primary aggregation factor in the hyphae of *A*. *nidulans*. In comparison with the parental strain, the α-1,3-glucan-deficient mutant of *A*. *nidulans* showed the same levels of radial growth and conidiation on agar plates [27], and its mycelial weight in liquid culture was higher [67]. In the *kuro* (black) koji mold *Aspergillus luchuensis*, although an *agsE* disruption mutant formed hyphal pellets, their average diameter was lower than in the wild-type strain in shake-flask culture [68]. The hyphae of wild-type *A*. *oryzae* form pellets similar to those of wild-type *A*. *nidulans* in shake-flask culture [27, 67]. Miyazawa et al. [67] reported that a triple disruption strain of *A*. *oryzae* defective in α-1,3-glucan synthase genes (Δ*agsA*Δ*agsB*Δ*agsC*) lacked α-1,3-glucan and the diameter of its pellets was decreased by 60% compared to that of the wild-type strain in shake-flask culture. The recombinant protein productivity of the Δ*agsA*Δ*agsB*Δ*agsC* mutant in flask culture was twice that of the wild-type strain [67]. Recently, Jeennor et al. [69] reported that an *A*. *oryzae* mutant defective in the *ags1* (*agsB*) gene showed an increase in lipid productivity in submerged flask and 5-L reactor cultivation in comparison with the parental strain. Recently developed software enables automatic characterization of hyphal macromorphology including hyphal area, pellet diameter, aspect ratio, and solidity [70]. Applying this software for characterization of α-1,3-glucan mutant hyphae could be a new approach to understand the relationship between fungal cell wall composition and morphogenesis.

Although the amounts of α-1,3-glucan affect the macromorphology of *Aspergillus* species, the relationship between the chemical structure of α-1,3-glucan and hyphal aggregation is less well understood. Miyazawa et al. [71] constructed *A*. *nidulans* strains overexpressing the α-1,3-glucan synthase genes *agsA* or *agsB* and found that the hyphae aggregated tightly in the *agsB*-overexpressing strain (as in the wild-type strain), but loosely in the *agsA*-overexpressing strain in shake-flask culture. Several chemical analyses revealed that the peak molecular mass of α-1,3-glucan was 372 ± 47 kDa in the *agsB*-overexpressing strain and 1480 ± 80 kDa in the *agsA*-overexpressing strain [71]. Fluorescent labeling with α-1,3-glucan-binding domain–fused GFP revealed that α-1,3-glucan is located in the outer layer of the cell wall in the wild-type and *agsB*-overexpressing strains, but in the inner layer in the *agsA*-overexpressing strain [71]. These results suggest that the degree of hyphal aggregation depends not only on the amount of α-1,3-glucan but also on its molecular mass and localization in the cell wall. The mechanism underlying different distribution of α-1,3-glucan in the cell wall according to its molecular mass is unknown, but one possible explanation is as follows. α-1,3-Glucan is synthesized on the plasma membrane at the hyphal tip. Larger α-1,3-glucan is less soluble than that with smaller molecular mass, and larger molecules are more readily insolubilized and immobilized right after their biosynthesis than are smaller molecules. α-1,3-Glucan from disruption strains defective in *amyG*, which encodes intracellular α-amylase related to biosynthesis of α-1,3-glucan, had smaller molecular mass than that of α-1,3-glucan from the wild type, and was localized in the outer layer of the cell wall [71], consistent with this possibility.

### Galactosaminogalactan

GAG was first isolated from *A*. *nidulans* culture supernatant in 1970 [72], and from *A*. *niger* cell wall material in 1976 [73]. The presence of GAG was also reported in the culture supernatant from *Penicillium frequentans* in 1988 [74]. GAG was reported as one of the components of ECM in *A*. *fumigatus* in 2010 by Loussert et al. [24], and then Fontaine et al. [75] determined its detailed chemical structure. GAG is a heteropolysaccharide composed of galactopyranose and GalNAc with linear α-1,4-linkage; GalNAc residues are partially deacetylated to galactosamine [75]. Lee et al. [76] reported that the ratio of galactopyranose to GalNAc differs among *Aspergillus* species; the *A*. *fumigatus* GAG is composed mostly of GalNAc (> 90%), but GalNAc content is lower (70%) in *A*. *nidulans*. The GalNAc-rich GAG is strongly bound to the cell wall [76]. Deacetylation of GAG is important for the adhesion of *A*. *fumigatus* hyphae on negatively charged surfaces [77]. It was suggested that the transcription factors StuA and MedA regulate GAG biosynthetic genes in *A*. *fumigatus* [77, 78]. GAG is synthesized by a protein complex encoded by five clustered genes (*gtb3*, *agd3*, *ega3*, *sph3*, and *uge3* in *A*. *fumigatus*) [77]. GAG is a biofilm component, and the absence of GAG causes a loss of crystal violet dyability [28, 77, 79]. GAG has an immunosuppressive effect and favors infection with *A*. *fumigatus* [75]. Hyphal β-1,3-glucan is scarcely exposed because of masking by hypha-associated GAG, resulting in impaired dectin-1 (β-1,3-glucan receptor)-mediated recognition by mammalian dendritic cells and macrophages [79]. Treatment of whole blood with purified soluble GAG increased the numbers of apoptotic neutrophils [75].

**Fig. 1:**
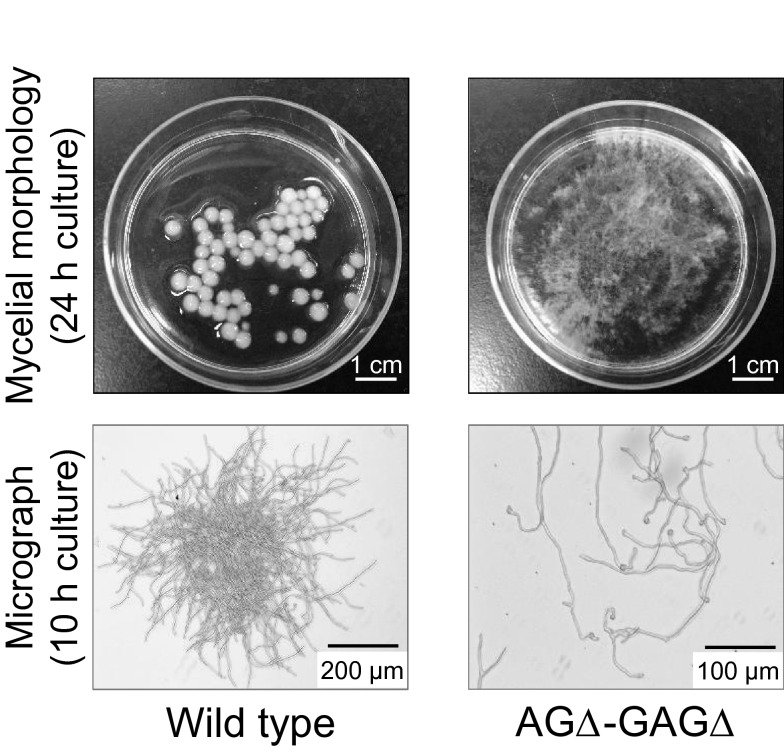
Macromorphology of wild-type *Aspergillus oryzae* and a double mutant deficient in α-1,3-glucan and galactosaminogalactan (AGΔ-GAGΔ). Upper images, mycelial morphology of both strains cultured for 24 h in an Erlenmeyer flask. Lower images, the hyphae of both strains cultured for 10 h observed under an inverted microscope. Conidia (1.0 × 10^5^/mL) of each strain were inoculated into 50 mL of YPD (2% peptone, 1% yeast extract, 2% glucose) medium in a 200-mL Erlenmeyer flask and rotated at 120 rpm at 30 °C

Although the hyphae of an α-1,3-glucan-deficient mutant of *A*. *oryzae* form smaller pellets than those formed by wild-type hyphae in shake-flask culture [67], they are not dispersed [27]. Miyazawa et al. [28] disrupted the *sphZ* and *ugeZ* genes (orthologous to *sph3* and *uge3* of *A*. *fumigatus*, respectively) in the α-1,3-glucan-deficient mutant of *A*. *oryzae* (AGΔ-GAGΔ strain), which resulted in complete loss of GAG from the cell wall and culture supernatant and complete dispersion of the hyphae in shake-flask culture (Fig. 1). These results suggest that GAG contributes to hyphal aggregation in *A*. *oryzae*, in addition to α-1,3-glucan. Interestingly, disruption of the *sphZ* and *ugeZ* genes in the wild-type strain resulted in larger aggregates than in the wild type [28]; the cause of this phenomenon remains unclear. On agar plates, growth and morphology (i.e. branches and septa) of disruptants of GAG biosynthetic genes are similar to those of the parental strain [28, 79], suggesting that GAG is not essential under these conditions. In vitro aggregation assay using partially purified GAG revealed that GAG directly aggregates *A*. *oryzae* hyphae, and that deacetylation of GalNAc residues in GAG is important for hyphal aggregation [28]. The AGΔ-GAGΔ strain of *A*. *oryzae*, which is deficient in both α-1,3-glucan and GAG, showed significantly higher recombinant protein productivity compared to those of the wild type and a mutant deficient in α-1,3-glucan only (AGΔ strain) in submerged cultivation [28]. For the future use of the double mutant for industrial production, further evaluation of its protein and secondary metabolite productivity is ongoing in our laboratories.

### Contribution of the chemical properties of α-1,3-glucan and GAG to hyphal aggregation

As discussed in the previous subsections, α-1,3-glucan and/or GAG are important for hyphal aggregation in submerged culture of filamentous fungi, suggesting that the two polysaccharides contribute to hyphal pellet formation. The distribution of α-1,3-glucan and GAG biosynthetic genes among fungi is listed in Table 1. Lee et al. [77] reported that GAG biosynthetic genes are present in some Pezizomycotina (ascomycetes) such as *Aspergillus*, *Penicillium*, and *Botrytis*; among basidiomycetes, only the plant pathogenic yeast *Trichosporon asahii* has these genes. In contrast, α-1,3-glucan synthase genes are distributed among various ascomycetes and basidiomycetes. α-1,3-Glucan and/or GAG biosynthetic genes are present in most industrially relevant fungi, but are absent in *Rhizopus* and *Trichoderma* species. Therefore, controlling α-1,3-glucan and GAG in the cell wall could help to manage the pellet formation of most filamentous fungi and subsequently ensure efficient microbial production.

**Table 1:** Distribution of α-1,3-glucan and galactosaminogalactan (GAG) biosynthetic genes in fungi

Phylum	Subphylum	Species	α-1,3-Glucan	GAG
Mucoromycota	Mucoromycotina	*Mucor circinelloides*	–	–
		*Rhizopus oryzae*	–	–
Ascomycota	Taphrinomycotina	*Schizosaccharomyces pombe*	+	–
	Saccharomycotina	*Dekkera bruxellensis*	–	–
		*Candida albicans*	–	–
		*Kluyveromyces lactis*	–	–
		*Ashbya gossypii*	–	–
		*Zygosaccharomyces rouxii*	–	–
		*Saccharomyces cerevisiae*	–	–
		*Candida glabrata*	–	–
	Pezizomycotina	*Bipolaris maydis*	–	+
		*Histoplasma capsulatum*	+	–
		*Aspergillus fumigatus*	+	+
		*Aspergillus nidulans*	+	+
		*Aspergillus oryzae*	+	+
		*Aspergillus niger*	+	+
		*Penicillium chrysogenum*	+	+
		*Botrytis cinerea*	+	+
		*Fusarium graminearum*	–	–
		*Trichoderma reesei*	–	–
		*Neurospora crassa*	+	+
		*Sordaria macrospora*	+	+
Basidiomycota	Pucciniomycotina	*Rhodosporidium toruloides*	–	–
		*Puccinia graminis*	+	–
		*Wallemia sebi*	–	–
	Ustilaginomycotina	*Malassezia globosa*	–	–
		*Ustilago maydis*	–	–
	Agaricomycotina	*Trichosporon asahii*	+	+
		*Cryptococcus neoformans*	+	–
		*Tremella mesenterica*	+	–
		*Auricularia delicata*	+	–
		*Fomitiporia mediterranea*	+	–
		*Punctularia strigosozonata*	+	–
		*Stereum hirsutum*	–	–
		*Coniophora puteana*	–	–
		*Schizophyllum commune*	+	–
		*Coprinopsis cinerea*	+	–
		*Laccaria bicolor*	+	–

**Fig. 2:**
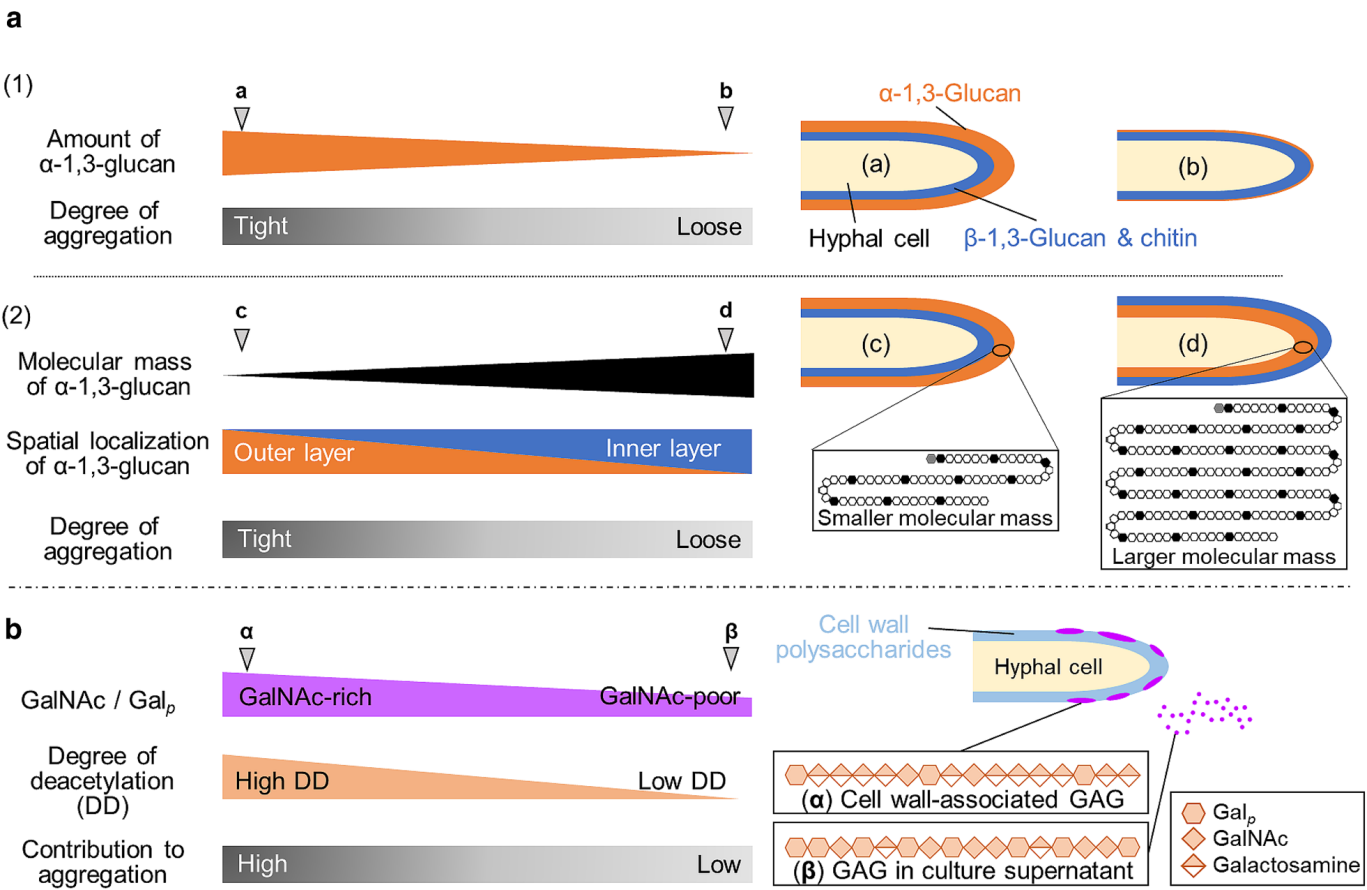
Speculative models for contribution of (A) α-1,3-glucan and (B) galactosaminogalactan (GAG) to hyphal aggregation in submerged culture of *Aspergillus* species. (**A**) (1) The amount of α-1,3-glucan contributes to the degree of hyphal aggregation. Hyphae rich in α-1,3-glucan (a) aggregate tightly, whereas α-1,3-glucan–poor hyphae (b) aggregate loosely or disperse. (2) Spatial localization of α-1,3-glucan also contributes to hyphal aggregation. α-1,3-Glucan with low molecular mass localizes in the outer layer of the cell wall (c), whereas α-1,3-glucan with high molecular mass localizes in the inner layer (d). (**B**) GAG that is GalNAc-rich and/or has a high degree of deacetylation of GalNAc residues is associated with the cell wall (α) and has a role in hyphal aggregation. In contrast, GAG that is GalNAc-poor and/or has a low degree of deacetylation of GalNAc residues has low adhesivity to the cell surface (β). Gal_*p*_, galactopyranose

The contribution of the chemical properties of α-1,3-glucan and GAG to hyphal aggregation is shown in Fig. 2. For α-1,3-glucan-mediated aggregation, the presence of α-1,3-glucan in the outer layer of the cell wall is important (Fig. 2a). Lack of α-1,3-glucan or its localization in the inner layer of the cell wall decreases the degree of hyphal aggregation. The primary regulatory factor for spatial localization of α-1,3-glucan in the cell wall is its molecular mass: the small molecules are localized in the outer layer and contribute to aggregation, and the large ones are localized in the inner layer. α-1,3-Glucan in the inner layer is covered with β-1,3-glucan and chitin, which reduces the degree of hyphal aggregation [71]. Disruption of *agd3* encoding a GalNAc deacetylase in *A. fumigatus* abolishes GAG deacetylation and leads to a loss of cell wall–associated GAG [77]. *N*-Acetylation of amino group in galactosamine of GAG (~ 98%) results in loss of the ability of *A*. *oryzae* hyphae to aggregate [28]. Thus, GAG-mediated hyphal aggregation seems to be related to the attachment of GAG to the cell surface. The ratio of GalNAc to galactopyranose and the degree of deacetylation of GalNAc residues in GAG are important for the adhesion of GAG to the cell surface (Fig. 2b). Amino groups of galactosamine residues generated by deacetylation of GalNAc residues are important for hydrogen bond formation between GAG molecules and between GAG and other cell wall components.

## Chemical structures and biosynthesis of α-1,3-glucan and galactosaminogalactan

Understanding the chemical structure and biosynthesis of α-1,3-glucan and GAG is important for the ability to regulate the degree of hyphal aggregation in liquid culture. Here we describe the current understanding of the chemical structure and the mechanism of biosynthesis of these polysaccharides.

### α-1,3-Glucan biosynthesis

The cell wall integrity signaling (CWIS) pathway regulates cell wall synthesis–related genes. This pathway has been studied in detail in the yeast *Saccharomyces cerevisiae* [80, 81]. The CWIS pathway is composed of sensor proteins, a GTPase, and a series of effectors. In particular, the protein kinase C–activated mitogen-activated protein (MAP) kinase cascade has been well analyzed [81]. The CWIS pathway is conserved in many filamentous fungi including *Aspergillus* species [16, 82, 83]. Damveld et al. [84] reported that α-1,3-glucan synthase genes are induced in the presence of a cell wall stress–inducing compound in *A. niger*. In *S*. *cerevisiae*, the expression of the genes involved in β-1,3-glucan and chitin synthesis is regulated through the MAP kinase Mpk1p of the CWIS pathway [85]. In contrast, α-1,3-glucan synthase genes are regulated through the MAP kinase MpkA, whereas β-1,3-glucan and chitin synthesis–related genes are regulated by other unknown signals, in *A*. *nidulans* [86].

The mechanism of α-1,3-glucan biosynthesis was first suggested from its chemical structure in the fission yeast *S. pombe* [87]. The α-1,3-glucan synthase gene *ags1* has been isolated using forward genetics, and the Ags1p protein is thought to be composed of intracellular, extracellular, and multitransmembrane domains [87]. The presence of Ags1p (Mok1p) in the membrane fraction was confirmed [88]. Grün et al. [89] analyzed the detailed chemical structure of α-glucan from *S. pombe* and found that its molecular mass is 42.6 ± 5.2 kDa and it is composed of 1,3-linked α-glucose (90%) and 1,4-linked α-glucose (10%). Each 1,4-linked α-glucan unit (≤ 12 residues) interconnects two 1,3-linked α-glucan units (1 unit ≈ 120 residues). 1,4-Linked α-glucan is also located at the reducing end of the α-glucan chain. Vos et al. [90] suggested that the intracellular domain is required for the biosynthesis of the α-1,4-glucan portion.

Choma et al. [91] reported the chemical structure of water-insoluble (alkali-soluble) glucan derived from the cell wall of *Aspergillus wentii*. This glucan is composed of 25 subunits, each consisting of 200 residues of 1,3-linked α-glucose; the subunits are separated by short spacers of 1,4-linked α-glucose residues [91]. The average molecular mass of this glucan is 850 kDa [91]. The structure of the alkali-soluble glucan from *A*. *nidulans* is similar to that from *A*. *wentii* [71]. Thus, α-1,3-glucan from *Aspergillus* has a larger molecular mass and larger number of repeated subunits in its chain than that from *S*. *pombe*.

Grün et al. [89] speculatively described the functional domains of α-1,3-glucan synthase of *S. pombe*. The intracellular domain contains an N-terminal Lys-rich region and C-terminal Ser-rich region, and a region with similarity to glycogen synthase and starch synthase, and is thought to have a role in the elongation of the α-1,3-glucan chain from UDP-glucose as a sugar donor. However, no direct evidence for the function of the intracellular domain has been reported. An amino acid substitution in the intracellular domain of *S*. *pombe* Ags1 prevents accumulation of α-1,4-glucan [90], implying that this domain might synthesize α-1,4-glucan. The extracellular domain shares sequence similarity with bacterial α-amylases of the glycosyl hydrolase family 13. The temperature-sensitive *S*. *pombe* mutant *ags1*-*1*^*ts*^, which has a G696S substitution in the extracellular domain, synthesizes immature shortened α-glucan without α-1,3-glucan interconnection [89], suggesting that the extracellular domain has a role in transglycosylation of exported α-1,3-glucan chains. The multitransmembrane domain is expected to contain 12 transmembrane helices that might form a pore-like structure. This domain is thought to export the α-1,3-glucan polymerized by the intracellular domain [89]. This domain structure is conserved in the α-1,3-glucan synthases from *Aspergillus* species [66].

In *Aspergillus* species, except for section Fumigati, the α-1,3-glucan synthase gene (*A*. *nidulans agsB* or its orthologue) is clustered with two α-amylase-encoding genes [66]. In *A*. *nidulans*, these are the intracellular α-amylase gene, *amyG*, and the gene coding for GPI-anchored α-amylase, *amyD*. Marion et al. [92] reported that the intracellular α-amylase from *H*. *capsulatum* (Amy1) is essential for biosynthesis of α-1,3-glucan. AmyA from *P*. *brasiliensis* seems to have a similar role [93]. Although fungal intracellular α-amylase is thought to synthesize 1,4-linked α-glucan as a primer or spacer structure for the α-1,3-glucan chain [92, 94], no direct evidence has been reported. van der Kaaij et al. [94] reported that AmyD from *A. niger* produces maltotriose from starch with low hydrolytic activity, and has no hydrolytic activity against glycogen or UDP-glucose, so the main substrate of AmyD in vivo remains unclear. Disruption of *amyG* in *A*. *nidulans* significantly decreases the amount of α-1,3-glucan [66, 71]. In addition, the molecular mass of α-1,3-glucan is significantly lower in the *amyG* disruptant than in wild-type *A*. *nidulans* and corresponds to only two α-1,3-glucan subunits [71]. In *Aspergillus* species, AmyG and its orthologues probably function in spacer synthesis, and another protein might synthesize the primer for the initiation of α-1,3-glucan polymerization.

*amyD* represses α-1,3-glucan biosynthesis in *A*. *nidulans* [66, 95]. He et al. [95] suggested that the mechanism of the decrease in the amount of cell wall α-1,3-glucan by AmyD differs from that by α-1,3-glucanase in *A. nidulans*. *Aspergillus niger* AgtA, encoded by a gene orthologous to *A*. *nidulans amyD*, uses maltopentaose or maltohexaose as substrates to produce maltooligosaccharides with a degree of polymerization of up to 28 [96]. AgtA uses maltose, nigerose, and nigerotriose as acceptor substrates [96].

In *H. capsulatum*, repression of the *UGP1* gene encoding UTP-glucose-1-phosphate uridylyltransferase (UDPGP) causes loss of cell wall α-1,3-glucan [92]. UDPGP converts glucose-1-phosphate and UTP to UDP-glucose, suggesting that UDP-glucose might be the substrate for α-1,3-glucan biosynthesis.

**Fig. 3:**
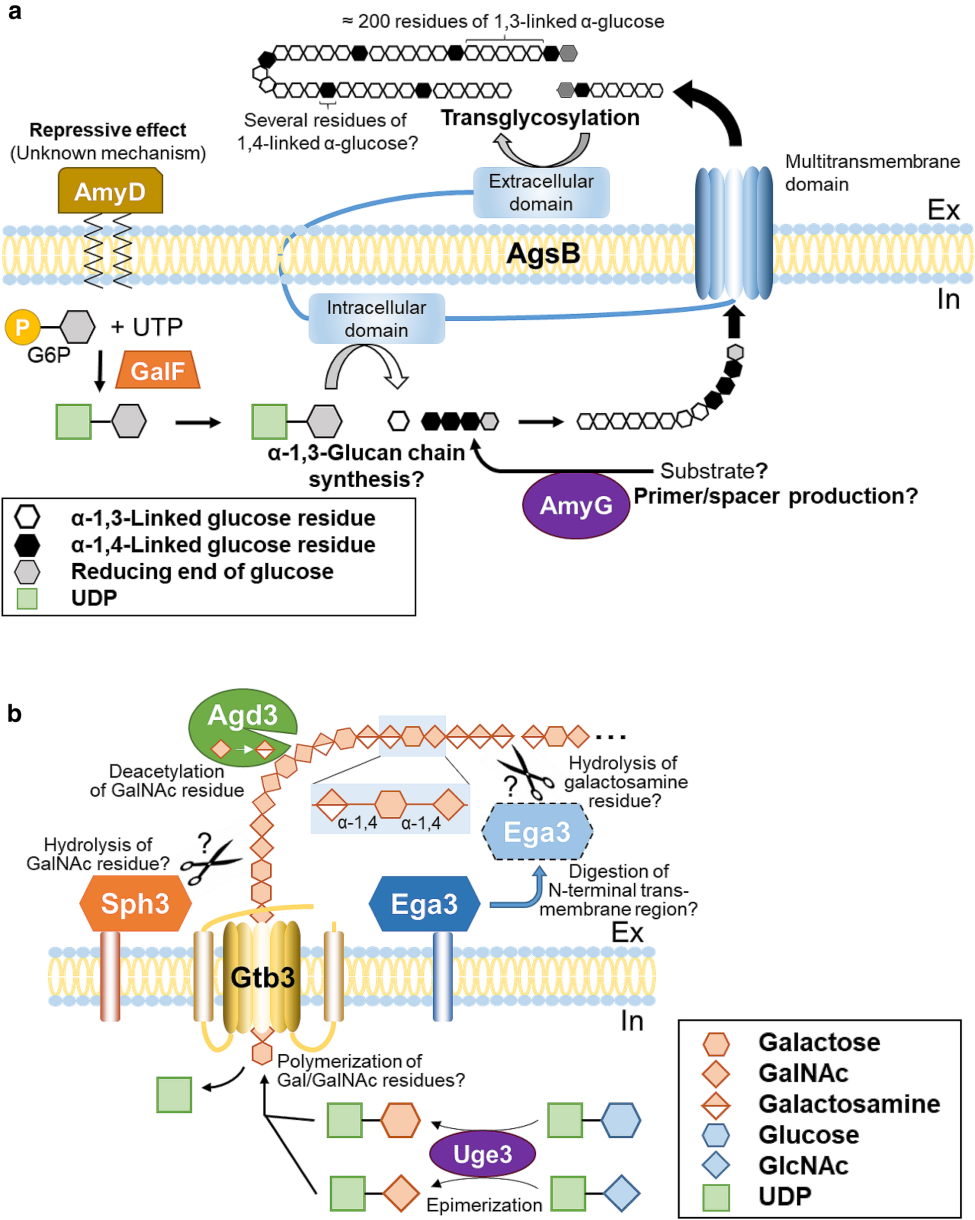
Speculative model for biosynthesis of (A) α-1,3-glucan and (B) galactosaminogalactan (GAG) in *Aspergillus* species. **A** AgsB, an α-1,3-glucan synthase, has three domains: extracellular, intracellular, and multitransmembrane. The likely substrate for α-1,3-glucan synthesis, UDP-glucose, is produced from glucose-6-phosphate and UTP by the UTP-glucose-1-phosphate uridylyltransferase GalF, which is encoded by a gene orthologous to *H*. *capsulatum UGP1*. Maltooligosaccharide produced by intracellular α-amylase, AmyG, might act as a primer for polymerization of glucose from UDP-glucose as the sugar donor; polymerization is performed by the intracellular domain of AgsB, resulting in a subunit composed of approximately 200 residues of 1,3-linked α-glucose with a short 1,4-linked α-glucose primer at its reducing end. The polymer synthesized by the intracellular domain is exported through a pore-like structure of the multitransmembrane domain. Then the extracellular domain catalyzes interconnection of several subunits, resulting in a mature α-1,3-glucan chain. A GPI-anchored α-amylase, AmyD, has a repressive effect on α-1,3-glucan biosynthesis, but the detailed mechanism of this effect remains unclear. G6P, glucose-6-phosphate. Protein names are for *A*. *nidulans* unless otherwise noted. **B** The UDP-glucose 4-epimerase Uge3 epimerizes UDP-glucose to UDP-galactopyranose and UDP-*N*-acetylglucosamine to UDP-*N*-acetylgalactosamine (GalNAc). UDP-galactopyranose and UDP-GalNAc are expected to be polymerized by Gtb3. The polymers are thought to be exported from the cell through a pore formed by Gtb3, and then the GalNAc residues are partially deacetylated by Agd3. The deacetylated polymer is mature GAG and either associates with the cell wall or is dissolved into the culture supernatant. Sph3 hydrolyzes the GalNAc residues owing to its endo-α-1,4-*N*-acetylgalactosaminidase activity. Ega3 might be released by digestion of its N-terminal transmembrane region, and might then hydrolyze the deacetylated GAG, but no direct evidence has been reported. Protein names are for *A*. *fumigatus*

Overall, the biosynthesis of α-1,3-glucan in *Aspergillus* species may proceed as follows (Fig. 3a). UDPGP synthesizes UDP-glucose from glucose-1-phosphate and UTP. Maltooligosaccharides produced by intracellular α-amylase might act as primers for polymerization catalyzed by the intracellular domain of α-1,3-glucan synthase. The intracellular domain catalyzes glucose polymerization from UDP-glucose as the sugar donor, resulting in synthesis of 1,3-linked α-glucan; these reactions produce a subunit composed of 1,3-linked α-glucan and the 1,4-linked primer at its reducing end. The subunit is exported to the outer side of the membrane through the multitransmembrane domain of α-1,3-glucan synthase, and two subunits are interconnected by its extracellular domain. The interconnection occurs several times, resulting in a mature α-1,3-glucan chain. AmyD represses α-1,3-glucan accumulation, but its function during the biosynthesis remains unclear. Further studies are needed to unveil the detailed mechanism underlying the biosynthesis of α-1,3-glucan.

### GAG biosynthesis

A GAG biosynthetic gene cluster has been identified in *A*. *fumigatus*, and the mechanism of GAG biosynthesis has been studied by analyzing the proteins encoded by the clustered genes [77]. The average molecular mass of GAG from *A*. *fumigatus* is approximately 100 kDa (range, 10–1000 kDa) [75]. Deacetylation of GalNAc residues depends on the deacetylase Agd3 in *A*. *fumigatus* [77]. GAG-dependent adhesion is controlled by the degree of GalNAc deacetylation [77]. The ratio of GalNAc to galactose is also important for hyphal aggregation [79]. Thus, localization of insolubilized GAG on the cell surface via deacetylated GalNAc residues is thought to be required for hyphal aggregation.

The detailed mechanism underlying GAG biosynthesis has been reported by Speth et al. [97] in *A*. *fumigatus*. Briefly, this mechanism includes: [1] UDP-galactose and UDP-GalNAc production by Uge3 in the cytoplasm; [2] polymerization of galactose and GalNAc and export of the polymer through the membrane by Gtb3; [3] deacetylation of the polymer by Agd3 to produce mature GAG (Fig. 3b). Bamford et al. [98] recently reported that Ega3 has endo-α-1,4-galactosaminidase activity, but the function of Ega3 during GAG biosynthesis is still unknown. The authors suggested that the N-terminal transmembrane region of Ega3 is cleaved, and then the mature Ega3 form is released from the membrane and hydrolyzes deacetylated GAG [98] (Fig. 3b). Sph3 shows endo-α-1,4-*N*-acetylgalactosaminidase activity [99, 100]. Although disruption of the *sph3* gene results in lack of GAG [99], the mechanism underlying this defect remains unclear. Although Gtb3 has not yet been characterized, it might contribute to polymerization of galactose and GalNAc, and to the outward translocation of the polymer through the pore-like region [77]. Sheppard and Howell [21] reported that the disruption of the *gtb3* gene in *A*. *fumigatus* resulted in the absence of GAG. We also confirmed the absence of GAG in the *A*. *oryzae* disruptant lacking the *gtbZ* gene (orthologous to *A*. *fumigatus gtb3*) (Miyazawa et al., unpublished results), suggesting that GtbZ is essential for GAG biosynthesis. Uge3 has epimerase activity that converts UDP-glucose into UDP-galactopyranose and UDP-*N*-acetylglucosamine into UDP-GalNAc [79, 101].

## Conclusions and future prospects

Control of hyphal aggregation is an issue that needs to be resolved in industrial production using filamentous fungi. We recently reported the contribution of α-1,3-glucan and GAG to hyphal aggregation in *A. oryzae*. Regulating the biosynthesis of these polysaccharides could help to optimize the degree of hyphal aggregation. Not only the amount of these polysaccharides but also their chemical structure affects hyphal aggregation. Some fungi such as *Trichoderma* species have no α-1,3-glucan or GAG biosynthetic gene cluster, implying that an unknown and unique polysaccharide or protein contributing to hyphal aggregation might be found. Recently, an outstanding technique for characterizing fungal pellet structure has been reported [102]. Understanding the relationship between cell wall polysaccharides and hyphal pellet formation by taking advantage of this new technology could contribute to the improvement of the productivity of cell factories that use filamentous fungi. To further increase the productivity of proteins and chemicals in industrial fermentation using filamentous fungi, understanding the mechanism of biosynthesis of cell wall polysaccharides and their biochemical properties is necessary.

## Data Availability

Not applicable.

## Abbreviations

CWIS: Cell wall integrity signaling
ECM: Extracellular matrix
GAG: Galactosaminogalactan
GalNAc: *N*-acetylgalactosamine
GPI: Glycosylphosphatidylinositol
MAP kinase: Mitogen-activated protein kinase
UDPGP: UTP-glucose-1-phosphate uridylyltransferase
